# Biomechanical and Psychological Predictors of Failure in the Air Force Physical Fitness Test

**DOI:** 10.3390/sports10040054

**Published:** 2022-04-06

**Authors:** Jeffrey Turner, Torrey Wagner, Brent Langhals

**Affiliations:** 1 Data Analytics Certificate Program, Graduate School of Engineering and Management, Air Force Institute of Technology, Wright-Patterson AFB, OH 45433, USA; jeffturner7@gmail.com (J.T.); brent.langhals@afit.edu (B.L.); 2 The Perduco Group (a LinQuest Company), Dayton, OH 45433, USA

**Keywords:** predictive modeling, military, risk management, physical fitness test, neural network

## Abstract

Physical fitness is a pillar of U.S. Air Force (USAF) readiness and ensures that Airmen can fulfill their assigned mission and be fit to deploy in any environment. The USAF assesses the fitness of service members on a periodic basis, and discharge can result from failed assessments. In this study, a 21-feature dataset was analyzed related to 223 active-duty Airmen who participated in a comprehensive mental and social health survey, body composition assessment, and physical performance battery. Graphical analysis revealed pass/fail trends related to body composition and obesity. Logistic regression and limited-capacity neural network algorithms were then applied to predict fitness test performance using these biomechanical and psychological variables. The logistic regression model achieved a high level of significance (*p* < 0.01) with an accuracy of 0.84 and AUC of 0.89 on the holdout dataset. This model yielded important inferences that Airmen with poor sleep quality, recent history of an injury, higher BMI, and low fitness satisfaction tend to be at greater risk for fitness test failure. The neural network model demonstrated the best performance with 0.93 accuracy and 0.97 AUC on the holdout dataset. This study is the first application of psychological features and neural networks to predict fitness test performance and obtained higher predictive accuracy than prior work. Accurate prediction of Airmen at risk of failing the USAF fitness test can enable early intervention and prevent workplace injury, absenteeism, inability to deploy, and attrition.

## 1. Introduction

The purpose of the Air Force Physical Fitness Test (APFT) is to promote year-round physical conditioning resulting in increased productivity, decreased absenteeism, and optimized readiness [1,2]. Failure to pass the APFT can directly impact mission capabilities, ability to deploy, workplace injuries, and attrition. The U.S. Army Public Health Service Center produces statistics annually for the US Army documenting the rising rates of inactivity, obesity, and Army Physical Fitness Test failures. The 2015 report identified that one in 20 active-duty Army Soldiers fail to pass their annual fitness test resulting in a three-fold increased risk of inability to deploy [3]. The report also noted that it costs the U.S. Army $137 million annually to recruit and train soldiers discharged due to test failure ($76,000 per recruit). An accurate predictive model could refer specific Airmen to targeted wellness initiatives through established installation human performance services, such as the Health Promotions Office, Fitness Facility, Aerospace Physiology, and Integrated Operational Support Teams [4,5].

There is a growing need for studies to evaluate predictors of military fitness test failure to address and reduce the impacts on health and deployment readiness [2]. One study investigated attrition in the civilian recruit population prior to and during basic military training and reviewed demographic, cognitive, mental health, physical health, and personal fitness [6]. While valuable for screening recruits, this study did not apply machine learning algorithms to predict APFT performance.

Three prior studies used machine learning algorithms to predict either test or test component performance, and they are summarized in Table 1. Orr et al. (2020) used age, height, weight, initial fitness performance, and course duration to predict fitness test failure in the Australian Army [7]. Sih and Negus (2016) used a time-series fitness and fatigue phenomenological model to predict Army Basic Combat Training two-mile run time, which is a component of the U.S. Army fitness assessment [8]. Finally, Allison, Knapick, and Sharp (2006) used age, height, weight, initial test results, test scores, education, number of dependents, and demographic data to predict U.S. Army fitness assessment failure [9].

The prior works evaluated demographic and biomechanical variables into their prediction models, but have not considered psychological and social variables as predictors, such as workplace sleepiness, burnout, traumatic stress, and social wellness. They also did not apply neural networks as a predictive algorithm. There is supporting evidence that psychological factors are associated with physical activity engagement [10], improved training effectiveness [11], and can predict dropout in military populations [12].

This study examines both biomechanical and psychological variables to predict fitness test failure, and focuses on the following research question: Using available biopsychosocial data, how well can classical machine learning and neural network algorithms predict failure in the Air Force Physical Fitness Test in a population of active-duty Airmen? It was hypothesized that combining biomechanical and psychosocial features would yield a better predictive model with included features that are significant (*α* ≤ 0.05), an area under the receiver operator characteristic curve (AUC) that improves upon the prior work in Table 1 (AUC ≥ 0.80), and accuracy > 0.90, which is consistent with other medical studies [13]. Additionally, it was hypothesized that applying a neural network model would yield better model performance than a logistic regression model.

## 2. Methods

This study was conducted on an existing dataset collected from a support squadron at a U.S. Air Force Base [14,15]. This support squadron was comprised of ~280 active-duty personnel. Data collection occurred across multiple days in February and July 2021. Advertisement for participation was conducted by word of mouth, squadron emails, and flyers. All potential participants were informed that the data collected would be anonymous and that the fitness testing would not be counted as their official diagnostic APFT. This was done to reduce fear of reprisal and boost participation. All data was collected as a part of the operational mission of an embedded, multidisciplinary health and wellness team [15]. Informed consent was collected on all participants and data was deidentified at the point of collection. The 223-sample dataset is summarized in Table 2 and includes features ranging from demographics, mental health surveys, fitness participation surveys, injury history surveys, physical performance measures, and body composition assessments.

### Data Understanding and Preparation

The data were prepared, processed, and analyzed using the Scikit-learn, Keras, and TensorFlow frameworks within Python version 3.7. The cross-industry standard process for data mining (CRISP-DM) was followed, with the phases of data understanding, data preparation, modeling, and evaluation [23].

An interview with the embedded health team that provided the data described the protocol of the testing day [15].

- Participants would start with the physical and mental health questionnaires, mental health questionnaires were proctored by a licensed clinical psychologist, immediately followed by a body composition assessment proctored by a registered dietitian.
- Next, the participants moved on to performing the Functional Movement Screen (FMS) proctored by a certified and credentialed athletic trainer. The FMS protocol is described well in the literature [21,22,24,25,26].
- The mental health questionnaires evaluated participant’s rating of wellbeing across multiple social domains [16], traumatic stress symptoms [17], sleepiness during the workday [18], and reported level of burnout [19].
- The physical questionnaires assessed (1) their six-month history of musculoskeletal injury and whether the member sought medical evaluation for that injury, (2) if the member sustained an injury within the last six months, and if it impacted their participation in physical activities, (3) whether or not they were currently on a duty-limiting medical profile, and (4) on a five-point Likert scale the member indicated their perceived satisfaction with their current fitness level.
- BMI, body fat percentage, and muscle mass percentage were assessed by using the InBody230 (InBody LTD, Seoul, Republic of Korea) bioelectrical impedance analyzer [20].
- The assessment concluded with the administration of the APFT, which is further described below.

The APFT is comprised of a timed 1.5-mile run, one minute of push-ups, and one minute of sit-ups; all components were performed on the same day of the collected measurements in accordance with the documented protocol within the Air Force manual for Fitness Testing [27]. The 1.5-mile timed run was performed outdoors on a standard 400-m track. The push-ups and sit-ups were performed indoors with options for a 1-inch pad and/or toe-bar. The APFT was performed and proctored by certified Air Force Physical Training Leaders. Raw APFT scores were collected, scored according to the manual, and then categorized into a binary category of either pass (≥75% composite score and passed all components) or fail (<75% composite score or failed a component).

**Figure 1:**
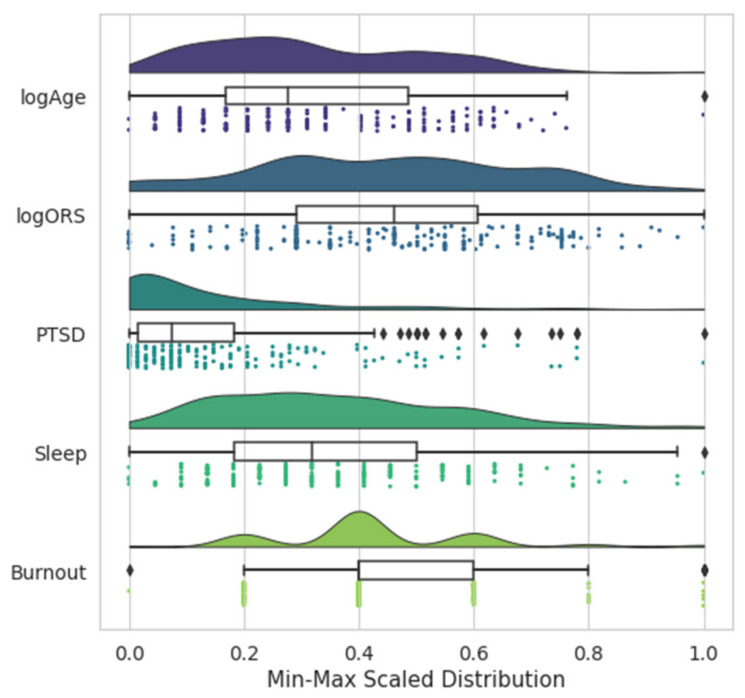

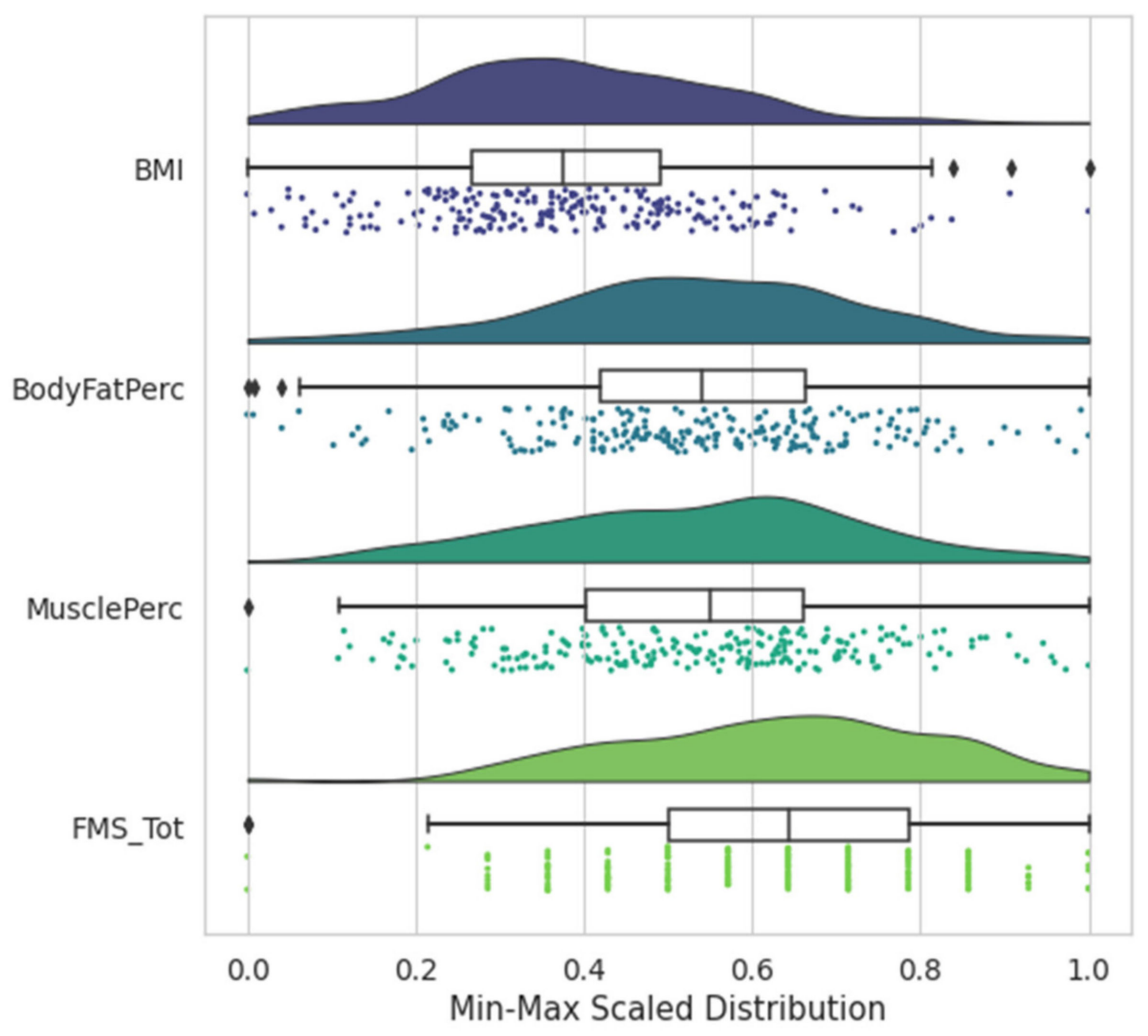
Selected numeric and ordinal categorical variable histograms. Acronyms and abbreviations: logAge, log-transformed age variable; logORS, log-transformed outcome rating scale variable; PTSD, post-traumatic stress disorder questionnaire; BMI, body mass index; FMS_Tot, functional movement screen composite score.

The binary dependent variable was whether the participant successfully passed all components of the APFT. This variable was unbalanced, as 70.4% of the participants passed the APFT. Preliminary modeling was conducted as a part of data preparation and selected categorical input variables were removed. Rank, Section, and Flight were removed as they each contained numerous categories and had a minimal influence on performance. The remaining numeric and ordinal categorical features were graphically analyzed for normality. Age and Outcome Rating Scale were identified as not normal and were log-transformed to increase their normality. While the Post-Traumatic Stress Disorder Checklist (PCL-5) feature was right-skewed, it was not transformed as various transformations did not create a normal distribution. The feature distributions are shown in the Figure 1 raincloud plot. To facilitate the visualization of the histograms, the features were temporarily min-max standardized prior to creating the figure.

## 3. Statistical Analysis

### 3.1. Metrics

In this analysis, the classification metrics of AUC, precision, recall, and accuracy are used to measure and compare the performance of the classical and neural network models. In the dataset, the positive class (1) is passing the APFT. In the case where an Airman failed the APFT, it is critical that a model prediction of passing (false positive) be avoided—this would result in the negative consequence of an at-risk Airmen not receiving assistance. As a result, a high value of precision is important to emphasize false positives and true positives. The confusion matrix false positives and the receiver operator characteristic (ROC) curve false positive rate were examined. Finally, precision and accuracy were selected as accurate predictors of true positives and to facilitate a comparison to prior work.

### 3.2. Classical Modeling

For the classical modeling part of the study, the python Logit logistic regression algorithm was used. The original dataset was split into a 70/30 train/test split for performance evaluation and overfitting monitoring. The split was stratified to ensure an equal percentage of pass/fail in each set.

Several feature selection approaches in logistic regression modeling were used to provide a robust comparison. Two models were created with a backwards stepwise feature selection approach, with an α = 0.05 *p*-value selection method. Recursive feature elimination (RFE) was also investigated, which fits the model and rank orders all features by importance, removing the weakest feature recursively. Lastly, the select-K-best method was used; this method selected the best predictors for the dependent variable using a Ψ^2^ function score. A summary of the logistic regression variations and their respective results are included in Table 3.

### 3.3. Neural Network Modeling

The Python 3.7 TensorFlow and Keras libraries were used to create neural networks to predict whether an Airman will pass their APFT. Binary cross-entropy was selected as the loss function since this was a binary classification problem. The Adaptive Moment Estimation (Adam) optimization algorithm was used as it is recommended for multi-layer networks [28,29]. The rectified linear unit (ReLU) activation function was used for all layers except for the final layer, which used a sigmoid activation function [30]. All non-output layers used L2 regularization with *λ* = 0.001. The same log transformations were applied as in the classical modeling, and then the data were z-score normalized.

One concern with this dataset was the relatively low ratio of 223 data points to 21 input variables. This concern was mitigated in two ways, and the first was to limit the capacity of the neural network. In the 1st International Conference on Neural Networks, Widrow proposed that the number of recommended datapoints *P* is the number of weights (neurons × (inputs + 1)) divided by the desired error level, according to the equation 1 below [31]. For our goal accuracy (0.90) the recommendation is 1 neuron for 21 inputs, and 4 neurons for 5 inputs. As a result, the capacity of the neural network was limited to within an order of magnitude of these recommendations, and potential overfitting was closely monitored.

(1) $$P=\frac{neurons\times \left(inputs+1\right)}{error}$$

The second method to address the low ratio of data points to input variables was to use a two-way dataset split with cross validation instead of the typical three-way train/test/holdout split used in neural networks. This maximized the number of datapoints available for training. The same 70/30 train/test stratified split was used as in the classical modeling, and three-fold cross validation was applied in the following manner:

- A multi-dimensional hyperparameter search (neurons, layers, learning rate, epochs, and batch size) was performed on the training dataset using three-fold cross validation. Total neuron count was limited by the Widrow recommendation [31].
- Overfitting was monitored by comparing the accuracy of each fold. Models with >5% inter-fold accuracy variance were not considered for selection.
- An optimal set of hyperparameters was determined by the highest mean fold accuracy of the remaining models.
- Using the optimal hyperparameters, the model was then retrained on the entire training dataset.
- The model was validated by measuring metrics on the holdout dataset.

This process was used twice: with all features and ≤10 total neurons and also on the four features from the best classical model (Sleep Score, BMI, Fitness Satisfaction, Physical Restriction) with ≤40 total neurons.

## 4. Results

**Figure 2:**
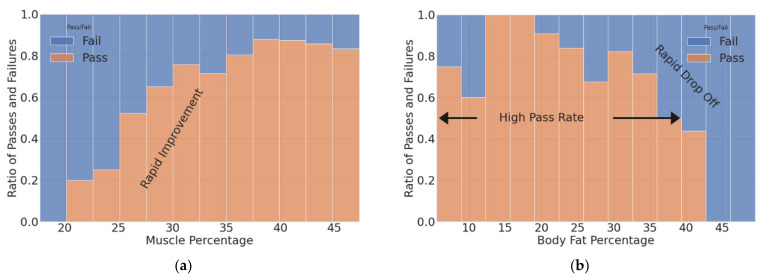
Graphical analysis of the ratio of passing/failing the APFT vs. (**a**) muscle percentage and (**b**) body fat percentage.

**Figure 3:**
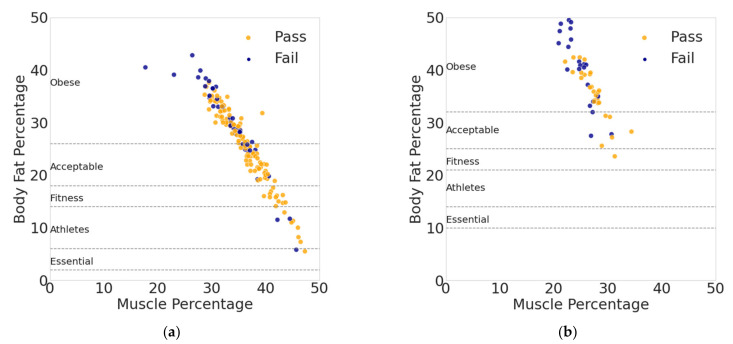
Gender differences in body composition and APFT pass/fail results for (**a**) male service members and (**b**) female service members. The figure is overlaid with biological gender-based body fat norms [32].

Graphical analysis revealed two trends that are highlighted in Figure 2 and Figure 3. The first trend is the influence of body composition on the ability to pass the APFT. As shown in Figure 2a, there was a clear correlation between increased muscle percentage and passing the APFT. This trend plateaued once the member’s muscle percentage reached 40%. As a corollary, Figure 2b shows an increase in the failure rate once the member’s body fat percentage increased past 20%.

The second trend is shown in Figure 3, which shows the relationship of obesity to passing the APFT. The figure is overlaid with biological gender-based body fat norms for men (Figure 3a) and women (Figure 3b) from the American Council on Exercise (ACE) [32]. A significant proportion of Airmen in the dataset are obese according to the ACE body fat norms, and a trend is apparent in Figure 3 that there is a much higher failure rate for those service members that are classified as obese. This is an important finding not only due to the correlation between higher body fat and failing the APFT, but also because of the health risks associated with obesity, such as heart disease and type 2 diabetes.

While 64% of overall Airmen were obese and 30% of overall Airmen failed the APFT, 82% of female Airmen were obese and 51% of female Airmen failed the APFT. Female Airmen also rated their satisfaction with their fitness lower than male Airmen: Only 21% of females rated their fitness satisfaction a 4 or higher (out of 5), while 41% of males did. While it is acknowledged this dataset is small and is not representative of the entire USAF, the trends regarding obesity and lack of fitness, especially for female Airmen, are noteworthy.

### 4.1. Model Results

The performance metrics are presented in this section for the five variations of classical logistic regression and three variations of neural network modeling. For comparison, metrics are also calculated for two trivial models: One that predicts randomly (*Chance*) and one that always predicts the majority class (*Always Predicts Pass*), also known as the no information rate model.

### 4.2. Classical Modeling

The 5 classical models developed in this work showed significance with a *p*-value < 0.01, and their AUC, precision, recall, and accuracy are presented in Table 3. For comparison, these performance metrics are also presented for the two trivial models, *Chance* and *Always Predicts Pass.* The goals of this study are also shown in the bottom row of the table.

The *four-feature p-value* model was found to be the best classical model, with a higher combination of accuracy, precision, recall, F1-score, and AUC compared to all other models. The following input variables were contained in this model: Sleep (*p*-value = 0.014), body mass index (BMI, *p*-value = 0.015), self-report of fitness satisfaction (FitSat, *p*-value < 0.01), and recent injury resulting in physical restriction (PhysRestr, *p*-value < 0.01). This model was better at predicting APFT pass (f1 = 0.89) than APFT failure (f1 = 0.77).

**Figure 4:**
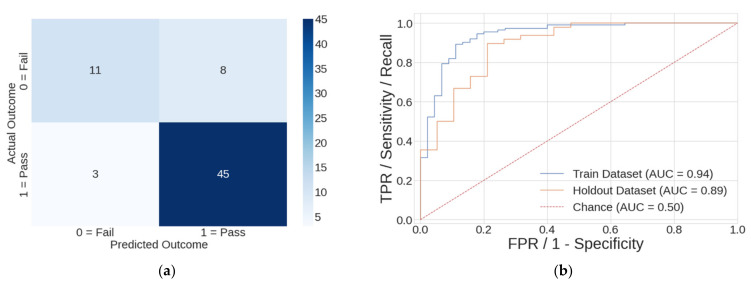
Performance graphs for the best *(four-feature p-value*) classical logistic regression model. (**a**) the confusion matrix resulting from the holdout dataset, where APFT Failure = 0 and APFT Pass = 1. (**b**) ROC curves from the training dataset, holdout dataset, and trivial *Chance* model. TPR: True positive rate; FPR: False positive rate.

For the *four-feature p-value* model, the confusion matrix from the holdout dataset is shown in Figure 4a along with the ROC curves from both the training and holdout datasets in Figure 4b. The confusion matrix showed that this model had eight false positives out of 19 APFT failures and three false negatives out of 48 APFT passing results. The ROC curve shows an acceptable 5% level of overfitting when comparing the AUC from the train and holdout datasets. While the selected classical model possessed good performance, it did not meet all the study goals.

### 4.3. Neural Network Modeling

Three neural network models are presented in this work: a baseline model on the entire dataset followed by models selected from multidimensional hyperparameter sweeps on two sets of input features. The baseline model was created with inputs to a single 10-neuron ReLU layer and a single-neuron sigmoid output layer. The first dataset for the hyperparameter sweeps was the entire 21-feature dataset, and the second dataset contained the 4 features from the best classical model: Sleep Score, BMI, Fitness Satisfaction, and Physical Restriction. The hyperparameter sweeps were performed using the GridSearchCV algorithm within the ranges shown in Table 4.

**Figure 5:**
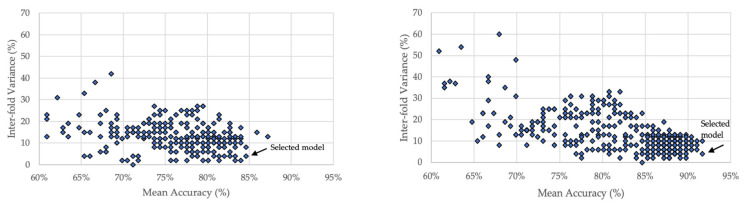
Families of models from the multidimensional hyperparameter sweeps on the full dataset (**left**), and the limited dataset (**right**). On each subfigure, an arrow indicates the best model.

For each family of models, metrics were collected and used to select the best model. As three-fold cross validation was used in all modeling, the accuracy for each fold was retained and used in two ways: (1) The mean accuracy was calculated across the three folds, and (2) the maximum variance was determined between the three folds. The best model was selected as the model with the highest mean accuracy that possessed the lowest inter-fold variance. These metrics for the families of models that resulted from the two variations is shown in Figure 5, along with an arrow that indicates the best model.

Using these criteria, the give neural network models that possessed the highest mean fold accuracy are presented in Table 5 for the full 21-feature dataset (top) and limited four-feature dataset (bottom). Their associated hyperparameters and inter-fold variance are shown as well. Bold text indicates the best models, which had an excellent combination of performance and limited complexity.

**Figure 6:**
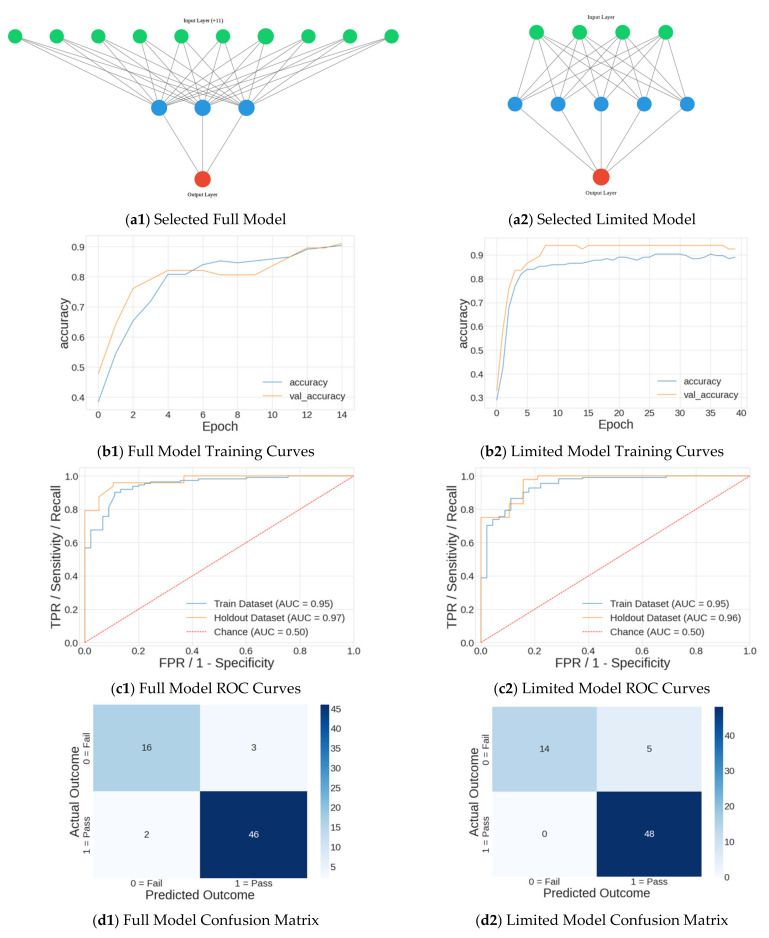
For the full model (**left**) and limited model (**right**): (**a1**,**a2**) Network structure where green are the input features, blue are neurons, and red is the output neuron; (**b1**,**b2**) training curves for the training (blue) and holdout (orange) datasets; (**c1**,**c2**) ROC curves and AUC for the training (blue) and holdout (orange) datasets; and (**d1**,**d2**) confusion matrix.

Concerning the selected “best” models, the neural network structure, accuracy vs. epoch training curves, ROC curves and AUC, and confusion matrix are shown in Figure 6. The left side of Figure 6 shows the model information for the full-dataset model, and the right side shows the limited-dataset model. There is a difference between the three-fold cross validation metrics and the final neural network performance metrics—for example, the accuracy for the full model in Table 5 (84.6%) is lower than the accuracy shown in Figure 6 panel b1 (93%). The authors propose this is due to the variation of training dataset size between the models. Three-fold cross validation (used for hyperparameter selection) used subdivisions of the training dataset, while the final model used those hyperparameters on the full training dataset.

The impacts of normalization on the neural network models were then investigated. The performance shown in Table 5 includes z-score normalization, and the hyperparameter search was repeated with normalization removed. Models were discarded that contained more that 10 neurons (full dataset model) or 40 neurons (limited dataset model). In this case the best full model with <5% inter-fold variance possessed a mean fold accuracy of 80.8%, which is 3.8% less than the model created from the z-score normalized dataset. The best limited model with <5% inter-fold variance possessed a mean fold accuracy of 84.0%, which is 7.7% less than the model created from the z-score normalized dataset.

The metrics for the neural network models presented in this work are summarized in Table 6 and were measured on the holdout dataset. Precision and recall are calculated as the weighted average between the majority and minority classes.

## 5. Discussion

In the current study, predictors of Air Force Physical Fitness Test failure were evaluated using logistic regression and limited-capacity neural networks. The models developed in this study demonstrated better performance than prior work to predict physical fitness test failure [7,8,9], and the neural network model achieved the highest level of performance. All models demonstrated an acceptable <5% level of overfitting between the training and holdout datasets.

The best logistic regression model was significant (*p*-value < 0.01), but it did not achieve the precision and accuracy goals of this study. While the logistic regression model underperformed the neural network, it yielded valuable inferences that poorer sleep quality, higher BMI, recent history of an injury, and a self-report of lower fitness satisfaction are potential indicators of APFT failure. Importantly, these features are modifiable with health promotion and workplace wellness interventions, which has the potential to change the likelihood of APFT failure.

A method of hyperparameter sweeps, regularization, and model selection enabled the neural network to outperform the logistic regression model. The 21-feature full dataset model yielded the best combination of AUC, precision, recall, and accuracy, as shown in Table 6. Notably, the near-identical performance from the four-feature limited dataset model (sleep quality, BMI, self-report of fitness satisfaction, and injury resulting in physical activity restriction) suggests that strong APFT prediction can be obtained with minimal data collection. This has the potential to enable wider implementation of this modeling approach as the full battery of tests requires a significant resource dedication. Future research investigating predictive modeling in sports science and injury prevention should consider limited-capacity neural network models.

If Air Force unit commanders and installation Health Promotion Offices implement unit-based surveys and body composition assessments of Airmen to assist in identification of individuals at greater risk of APFT failure, they could prioritize workplace interventions and installation health promotion assets to those individuals and units with the highest degree of need. These assets include the Health Promotions Office, Fitness Facility, Aerospace Physiology, and Integrated Operational Support Teams [4,5]. Changing from a reactive-based post-APFT failure model towards a proactive preventative model could improve retention and morale of military members.

Additionally, there is the potential for significant savings by preventing APFT failure. Successfully passing physical fitness testing and adhering to standardized exercise programming have been recognized as major contributors in noncombat injury risk reduction (33–45%) among active-duty members [33,34]. This is significant, as noncombat musculoskeletal injuries account for more than 80% of all injuries, 60% of limited duty days, and 65% of medically nondeployable active-duty members across the U.S. military [1]. Furthermore, the cost burden is immense for training and replacing warfighters discharged APFT failure, $137 million annually [3]. The USAF could save tens of thousands of dollars per Airman not lost to attrition through early identification of a servicemember likely to fail their APFT and early implementation of targeted wellness initiatives through established installation human performance services. Further research should be conducted evaluating similar biomechanical and psychosocial variables on more varied work centers in the USAF as well as other military branches.

A known limitation of this work is that participants were recruited from within a single USAF support squadron. This limits generalizability, and future research could include larger sample sizes and a greater variety of military occupations to validate the modeling approach. The difference in sample size (223) compared to the population size (280) is likely attributed to the optional nature of the study and the timing of data collection relative to shift work or approved leave.

## 6. Conclusions

The results of this study indicated that combining biomechanical and psychosocial variables yielded better prediction of failure on the USAF Fitness Test than previous work. In this work, biomechanical, psychological, and APFT performance data related to 223 active-duty Airmen was graphical analyzed to show pass/fail trends related to body composition and obesity. Multiple machine learning algorithms were then applied to predict fitness test performance using these variables. The logistic regression model achieved a high level of significance with an accuracy of 0.84 and AUC of 0.89 on the holdout dataset. This model showed that Airmen with poor sleep quality, recent history of an injury, higher BMI, and low fitness satisfaction tend to be at greater risk for fitness test failure. A limited-capacity neural network approach to predictive modeling yielded better performance than classical logistic regression—0.93 accuracy and 0.97 AUC on the holdout dataset. Given this greater understanding of the biopsychosocial variables that appear to predict failure in the USAF Fitness Test, the U.S. Department of Defense could achieve lower numbers of fitness test failures by mobilizing health promotion and workplace wellness campaigns targeting these identified variables.

## Figures and Tables

**Table 1:** Prior machine learning analyses in the prediction of military fitness test failures.

Description of Work	Method Used	Performance	Ref
Predict Fitness Assessment Failure in Australian Army	Classification, logistic regression.	AUC = 0.70	[7]
Predict U.S. Army Fitness Assessment 2-mile run time	Regression, phenomenological model.	R^2^ = 0.55–0.59	[8]
Predict U.S. Army Fitness Assessment Failure	Classification, logistic regression	AUC = 0.61–0.77 (F) AUC = 0.61–0.80 (M)	[9]

AUC: Area under the receiver operator characteristic (ROC) curve.

**Table 2:** Feature Summary Statistics.

Variable	Mean	Max	Std Dev	Data Distribution	Notes/Definition
Age *	28.15	59	6.60	~Log-Normal	---
Gender	0.25	1	0.43	Binary	0 = Male (174 members) 1 = Female (49 members)
ORS Total *	7.63	10	1.95	~Log-Normal	Outcome Rating Scale [16]
ORS Social	7.29	10	2.05	~Log-Normal	---
ORS Interpersonal	7.59	10	2.08	~Log-Normal	---
ORS Individual	7.35	10	2.02	~Log-Normal	---
PTSD	9.28	68	11.75	Right-skewed	Post-Traumatic Stress Disorder Checklist (PCL-5) [17]
Sleep	7.32	22	3.92	~Normal	--- [18]
Burnout	2.14	7	0.81	~Normal	--- [19]
InjuryEval	0.30	1	0.46	Binary	1 = Recent injury evaluated by provider
InjuryNoEval	0.12	1	0.33	Binary	1 = Recent injury not evaluated by provider
DLC	0.09	1	0.29	Binary	1 = Duty Limiting Condition
FitSat	3.32	5	0.81	Categorical	Fitness Satisfaction
PhysRestr	0.27	1	0.45	Binary	1 = Recent injury resulting in physical activity restriction
BMI	27.13	42.3	4.10	~Normal	Body Mass Index [20]
BodyFatPerc	0.29	0.49	0.82	~Normal	Body Fat Percentage [20]
MusclePerc	0.33	0.47	0.06	~Normal	Muscle Mass Percentage [20]
FMS_Shldr	0.12	1	0.33	Binary	1 = Functional Movement Screen (FMS) Shoulder Pain [21,22]
FMS_Ext	0.21	1	0.41	Binary	1 = FMS Low Back Pain [21,22]
FMS_Flex	0.06	1	0.23	Binary	1 = FMS Hip Pain [21,22]
FMS Total	14.28	20	2.60	~Normal	FMS Composite Score [21,22]

* indicates the variable will be log-transformed prior to modeling. The variable names are defined with references on the right side of the table.

**Table 3:** Logistic Regression Model Results as measured on the test/holdout dataset. The weighted average between the pass/fail classes are presented for precision and recall. The features used in each model are represented in the footnotes, and gray text indicates a trivial model.

Model	*p*-Value	AUC	Precision	Recall	Accuracy
Full	<0.01	0.82	0.79	0.79	0.79
5-feature *p*-value ^1^	<0.01	0.86	0.82	0.82	0.82
4-feature *p*-value ^2^	<0.01	0.89	0.83	0.84	0.84
Recursive feature elimination (RFE) ^3^	<0.01	0.87	0.75	0.75	0.75
Select K Best ^4^	<0.01	0.86	0.82	0.82	0.82
Chance	--	0.50	0.56	0.48	0.48
Always Predicts Pass	--	0.50	0.51	0.72	0.72
Goal	--	0.80	--	--	0.90

^1^: ‘Gender’, ‘Sleep’, ‘BMI’, ‘FitSat’, ‘PhysRestr’. ^2^: ‘Sleep’, ‘BMI’, ‘FitSat’, ‘PhysRestr’. ^3^: ‘Gender’, ‘InjuryNoEval’, ‘PhysRestr’, ‘FitSat’, ‘FMS_Flex’. ^4^: ‘MusclePerc’, ‘BodyFatPerc’, ‘FitSat’, ‘PhysRestr’, ‘DLC’.

**Table 4:** Neural network model hyperparameter search ranges.

Hyperparameter	Full Model Range	Limited Model Range
Neurons	2, 3, 4, 5, 10	1, 2, 3, 4, 5, 7, 9, 12
Hidden layers	0, 1, 2	0, 1, 2, 3
Batch size	16, 32	16, 32
Epochs	15, 20, 60, 100, 140	15, 20, 60, 100, 140
Learning rate	0.01, 0.001, 0.0005	0.01, 0.001, 0.0005

**Table 5:** Hyperparameters and metrics for the five best neural network models for the full 21-feature dataset (top) and the limited four-feature dataset (bottom). Bold text indicates the best model.

Dataset	Neurons	Layers	Learn Rate	Batch Size	Epochs	Mean Fold Accuracy (%)	Inter-Fold Variance (%)
Full 21 feature NN	10	1	0.001	16	140	87.2	13
	10	1	0.01	32	20	85.9	15
	**3**	**0**	**0.01**	**16**	**15**	**84.6**	**4**
	3	2	0.01	32	140	84.6	8
	5	2	0.01	16	20	84.0	2
Limited 4 feature NN	**5**	**0**	**0.01**	**16**	**40**	**91.7**	**4**
	3	1	0.01	16	100	91.7	10
	3	0	0.01	16	60	91.0	6
	40	1	0.001	32	60	91.0	10
	5	0	0.01	32	60	91.0	8

**Table 6:** Neural network modeling results as measured on the holdout dataset. The weighted average between the pass/fail classes are presented for precision and recall.

Model	AUC	Precision	Recall	Accuracy
Baseline	0.94	0.89	0.90	0.90
Full 21-input model	0.97	0.92	0.93	0.93
Limited 4-input model	0.96	0.93	0.93	0.93
Goal	0.80	--	--	0.90

## Data Availability

The dataset is publicly available [14].

## Authors’ Note

The views and opinions of authors expressed herein do not necessarily state or reflect those of the U.S. Government or any agency thereof. Reference to specific commercial products does not constitute or imply its endorsement, recommendation, or favoring by the U.S. Government. The authors declare this is a work of the U.S. Government and is not subject to copyright protections in the United States. This article was cleared with case numbers 88ABW-2021-0947 and MSC/PA-2021-0418.

## References

[1] Molloy J., Pendergrass T., Lee I., Hauret K., Chervak M., Rhon D. (2020). Musculoskeletal Injuries and United States Army Readiness. Part II: Management Challenges and Risk Mitigation Initiatives. Mil. Med..

[2] Orr R., Sakurai T., Scott J., Movshovich J., Dawes J.J., Lockie R., Schram B. (2021). The Use of Fitness Testing to Predict Occupational Performance in Tactical Personnel: A Critical Review. Int. J. Environ. Res. Public Health.

[3] U.S. Army (2015). Health of the Force.

[4] Baicker K., Cutler D., Song Z. (2010). Workplace wellness programs can generate savings. Health Aff..

[5] Gill D.L., Hammond C.C., Reifsteck E.J., Jehu C.M., Williams R.A., Adams M.M., Lange E.H., Becofsky K., Rodriguez E., Shang Y.-T. (2013). Physical Activity and Quality of Life. J. Prev. Med. Public Health.

[6] Knapik J., Darakjy S., Jones B., Hauret K., Piskator G. (2004). A Review of the Literature on Attrition from the Military Services: Risk Factors and Strategies to Reduce Attrition.

[7] Orr R., Cohen B., Allison S., Bulathsinhala L., Zambraski E., Jaffrey M. (2020). Models to predict injury, physical fitness failure and attrition in recruit training: A retrospective cohort study. Mil. Med. Res..

[8] Sih B., Negus C. (2016). Physical Training Outcome Predictions with Biomechanics, Part I: Army Physical Fitness Test Modeling. Mil. Med..

[9] Allison S., Knapik J., Sharp M. (2006). Preliminary Derivation of Test Item Clusters for Predicting Injuries, Poor Physical Performance, and Overall Attrition in Basic Combat Training.

[10] Ahn H., Kim Y., Jeong J., So Y. (2020). Physical Fitness Level and Mood State Changes in Basic Military Training. Int. J. Environ. Res. Public Health.

[11] Cardenas D., Madinabeitia I., Alarcon F., Perales J.C. (2020). Does Emotion Regulation Predict Gains in Exercise-Induced Fitness? A Prospective Mixed-Effects Study with Elite Helicopter Pilots. Int. J. Environ. Res. Public Health.

[12] Vaara J.P., Eranen L., Ojanen T., Pihlainen K., Nykanen T., Kallinen K., Heikkinen R., Kryolainen H. (2020). Can Physiological and Psychological Factors Predict Dropout from Intense 10-Day Winter Military Survival Training?. Int. J. Environ. Res. Public Health.

[13] Swets J. (1988). Measuring the accuracy of diagnostic systems. Science.

[14] Wagner T., Langhals B., Turner J. (2022). Dataset: Biomechanical & Psychological Predictors of Failure in the Air Force Physical Fitness Test. Mendeley Data.

[15] Caffrey Y. (2021). Personal communication.

[16] Miller S., Duncan B., Brown J., Sparks J., Claud D. (2003). The Outcome Rating Scale: A Preliminary Study of the Reliability, Validity, and Feaibility of a Brief Visual Analog Measure. J. Brief Ther..

[17] Wortmann J., Jordan A., Weathers F., Resick P., Dondanville K., Hall-Clark B., Foa E., Young-McCaughan S., Yarvis J., Hembree E. (2016). Pyschometric analysis of the PTSD Checklist-5 (PCL-5) among treatment-seeking military service members. Pyschol. Assess..

[18] Johns M. (1991). A new method for measuring daytime sleepiness: The Epworth sleepiness scale. Sleep.

[19] Hansen V., Pit S. (2016). The Single Item Burnout Measure is a Psychometrically Sound Screening Tool for Occupational Burnout. Health Scope.

[20] McLester B., Nickerson K., McLester J. (2020). Reliability and Agreement of Various InBody Body Composition Analyzers as Compared to Dual-Energy X-Ray Absorptiometry in Healthy Men and Women. J. Clin. Densitom..

[21] Bock K., Orr R. (2015). Use of the Functional Movement Screen in a Tactical Population: A Review. J. Mil. Veteran Health.

[22] Kollock R., Lyons M., Sanders G., Hale D. (2019). The effectiveness of the functional movement screen in determining injury risk in tactical occupations. Ind. Health.

[23] IBM Corporation (2011). IBM SPSS Modeler CRISP-DM Guide.

[24] Bonazza N., Smuin D., Onks C., Silvis M., Dhawan A. (2017). Reliability, Validity, and Injury Predictive Value of the Functional Movement Screen: A Systematic Review and Meta-analysis. Am. J. Sports Med..

[25] Teyhen D., Shaffer S., Lorenson C., Halfpap J., Donofry D., Walker M., Dugan J., Childs J. (2012). The Functional Movement Screen: A reliability study. J. Orthop. Sports Phys. Ther..

[26] Cuchna J., Hoch M., Hoch J. (2016). The interrater and intrarater reliability of the functional movement screen: A systematic review with meta-analysis. Phys. Ther. Sport.

[27] U.S. Air Force (2020). Air Force Manual 36-2905 Air Force Physical Fitness Program.

[28] Ruder S. (2016). An overview of gradient descent optimization algorithms. arXiv.

[29] Li K., Malik J. (2017). Learning to Optimize Neural Nets. arXiv.

[30] Goodfellow I., Bengio Y., Courville A. (2017). Deep Learning.

[31] Widrow B. ADALINE and MADALINE. Proceedings of the 1st International Conference on Neural Networks.

[32] American Council on Exercise (2021). Percent Body Fat Calculator: Skinfold Method. https://www.acefitness.org/education-and-resources/lifestyle/tools-calculators/percent-body-fat-calculator/.

[33] Knapik J., Rieger W., Palkoska F., van Camp S., Darakjy S. (2009). United States Army physical readiness training: Rationale and evaluation of the physical training doctrine. J. Strength Cond. Res..

[34] Molloy J. (2016). Factors influencing running-related musculoskeletal injury risk among U.S. military recruits. Mil. Med..

